# Messina: A Novel Analysis Tool to Identify Biologically Relevant Molecules in Disease

**DOI:** 10.1371/journal.pone.0005337

**Published:** 2009-04-28

**Authors:** Mark Pinese, Christopher J. Scarlett, James G. Kench, Emily K. Colvin, Davendra Segara, Susan M. Henshall, Robert L. Sutherland, Andrew V. Biankin

**Affiliations:** 1 Cancer Research Program, Garvan Institute of Medical Research, Darlinghurst, New South Wales, Australia; 2 Central Clinical School, University of Sydney, Camperdown, New South Wales, Australia; 3 Department of Anatomical Pathology, Royal Prince Alfred Hospital, Camperdown, New South Wales, Australia; 4 Division of Surgery, Bankstown Hospital, Bankstown, New South Wales, Australia; Swiss Federal Institute of Technology Lausanne, Switzerland

## Abstract

**Background:**

Morphologically similar cancers display heterogeneous patterns of molecular aberrations and follow substantially different clinical courses. This diversity has become the basis for the definition of molecular phenotypes, with significant implications for therapy. Microarray or proteomic expression profiling is conventionally employed to identify disease-associated genes, however, traditional approaches for the analysis of profiling experiments may miss molecular aberrations which define biologically relevant subtypes.

**Methodology/Principal Findings:**

Here we present Messina, a method that can identify those genes that only sometimes show aberrant expression in cancer. We demonstrate with simulated data that Messina is highly sensitive and specific when used to identify genes which are aberrantly expressed in only a proportion of cancers, and compare Messina to contemporary analysis techniques. We illustrate Messina by using it to detect the aberrant expression of a gene that may play an important role in pancreatic cancer.

**Conclusions/Significance:**

Messina allows the detection of genes with profiles typical of markers of molecular subtype, and complements existing methods to assist the identification of such markers. Messina is applicable to any global expression profiling data, and to allow its easy application has been packaged into a freely-available stand-alone software package.

## Introduction

Key molecular events in the development of disease are usually not ubiquitous, leading to instances of disease with similar morphological characteristics often having substantially disparate molecular phenotypes. This heterogeneity, far from being an unimportant epiphenomenon of disease development, has proven important in therapy: major advances in cancer therapeutics and patient outcomes have been achieved through the study and targeting of specific molecules and molecular mechanisms such as the estrogen receptor, and HER2/neu, aberrant expression of which occurs in approximately 30% and 25% of breast cancers, respectively [1], [2], [3]. Thus inconsistent molecular aberrations, which do not occur in every case of a disease, are biologically and clinically relevant and have proven useful in the development of effective therapies.

Traditional analysis methods for global gene expression data are poorly suited for the identification of genes that are aberrantly expressed at low frequency, and will fail to find genes that show aberrant expression in only a small subset of samples. This limitation in conventional techniques is gaining recognition, and new analyses of cancer datasets searching for genes with low frequency aberrant expression have produced novel insights into disease [4], [5].

Here we present Messina, a novel technique that identifies genes with lower frequencies of aberrant expression in disease. In contrast to currently available outlier detection methods (e.g. [4], [6], [7]), Messina can smoothly vary between identifying consistent differences, as in traditional approaches, to selecting the low frequency outliers found by current outlier detection techniques. This flexibility allows prior biological knowledge about the expected frequency of aberrant gene expression to inform the analysis, and enables the user to more specifically identify the genes of interest. Messina has its roots in machine learning theory, and its results can be directly used to implement robust single-gene classifiers separating case and control sample groups with user-supplied minimum sensitivity and specificity. In our work this has been useful in identifying lead target proteins for the development of radioactive tracers for disease diagnosis and localisation, but is generally applicable to any diagnostic problem for which the number of genes that can be measured is strictly limited. This paper describes the implementation, performance and validation of Messina compared to current commonly-used techniques, and demonstrates its utility in the detection of inconsistent differential expression in a case-control setting.

## Results

Messina is an algorithm for constructing classifiers capable of separating two sample groups (eg. cancer and normal tissue) on the basis of the expression level of a single gene. Classifiers (and thus genes) which can be used to separate the two sample groups are reported to the user as the final result of the algorithm.

Although Messina is fundamentally a classifier, its primary use is to identify genes with low frequency aberrant expression, such as markers of molecular subtype, thus complementing existing approaches that identify genes that show either consistent or outlier profiles. Messina identifies genes with low frequency aberrant expression by allowing the analyst to specify minimum sensitivity (the fraction of case samples that are placed into the case group by the classifier) and specificity (the fraction of control samples that are classified as being in the control group) constraints that classifiers must satisfy. The sensitivity can be considered to reflect the proportion of case samples with ‘case-like’ expression levels, and the specificity the proportion of control samples with ‘control-like’ expression. By modifying these two inputs the analyst can tune Messina to identify genes with particular profiles of expression across the spectrum from highly consistent differences (sensitivity and specificity both high), to genes detected by current outlier techniques (sensitivity low, specificity high). This link between classifier performance and low frequency aberrant expression is further developed in Methods S1.

### Messina selectively identifies genes of interest

We evaluated Messina's ability to detect genes that display aberrant expression in at least a user-defined proportion of samples using a series of simulation experiments which model genes with known frequencies of differential expression. These experiments examined how accurately Messina identified only those genes which satisfied its input performance constraints across a range of simulated experimental conditions. The selectivity of a simple t test was also examined to contrast its performance with Messina.

We generated simulated case-control expression data that spanned a range of sample sizes, differential expression magnitude (the difference between case and control sample distribution means), and differential expression degree. We defined differential expression degree as the fraction of simulated case samples which were drawn from the case distribution rather than the control distribution; this was varied in order to examine performance under conditions of inconsistent differential expression. Conventional analyses assume degree is equal to 1; values lower than 1 indicate that some case samples display control-like expression levels, and are indicative of low frequency aberrant expression. Under the conditions of the simulation, the simulated gene's differential expression degree was equal to the theoretical optimal sensitivity of a classifier based upon that gene. Therefore, an ideal gene detection response would be zero detection if the simulated gene's degree was less than the supplied sensitivity cutoff, and full detection otherwise.

**Figure 1:**
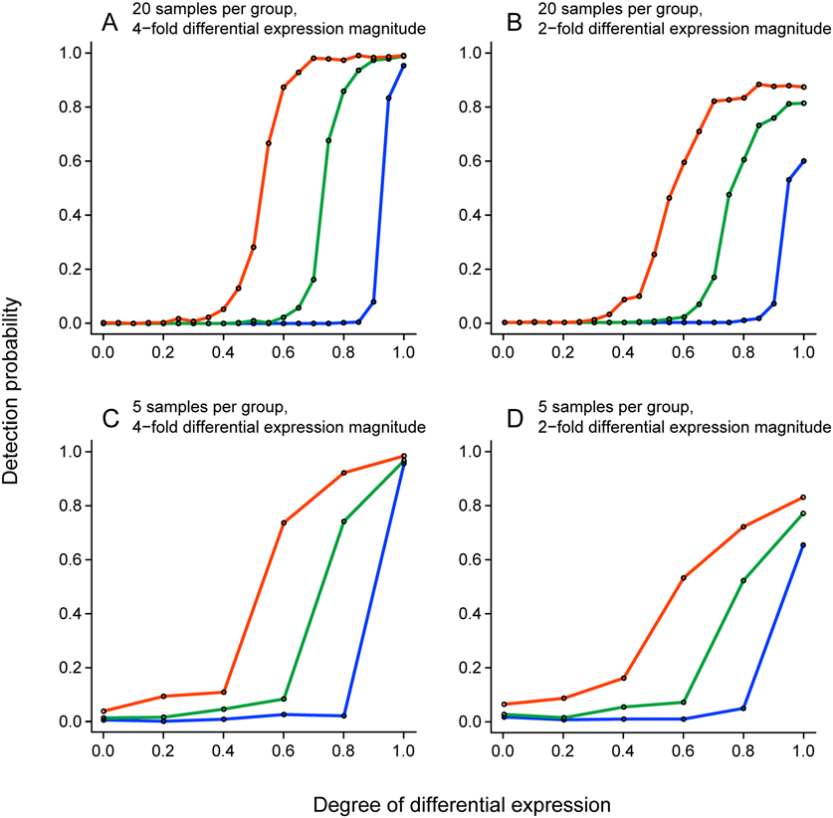
Messina detection performance. Each plot displays the probability that a gene will be detected by Messina, as a function of the gene's degree of differential expression, for three different sensitivity cutoffs: 50% (orange), 70% (green) and 90% (blue). Under these simulation conditions, an ideal response is zero detection for degrees under the sensitivity cutoff, and complete detection for degrees at or above the sensitivity cutoff. In all cases, Messina's specificity cutoff was set to 90%.

In all sample size scenarios, Messina detected only those genes with degrees of differential expression near to or exceeding its supplied sensitivity threshold (Figure 1). For genes with a small magnitude of differential expression (one log_2_ unit), Messina's detection efficiency reached a plateau at approximately 80% (Figure 1b,d), however for genes with a four-fold change and above Messina's detection efficiency rapidly approached 100% with increasing degree of differential expression, even in the case of only five samples per group (Figure 1a,c).

**Figure 2:**
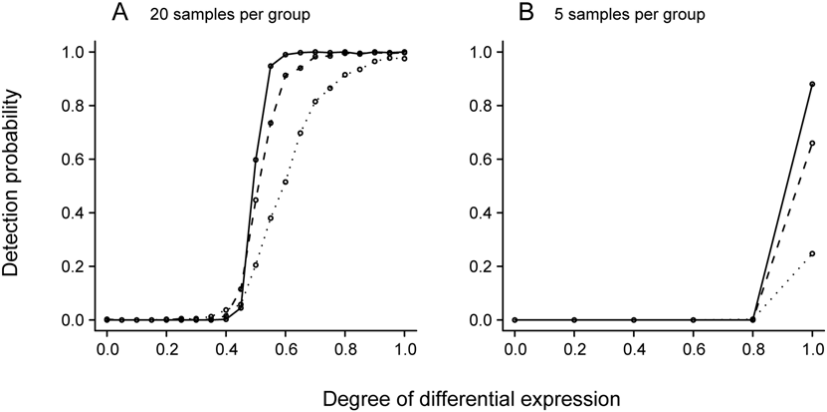
t test detection performance. As per Figure 1, each plot displays the probability of a gene being detected as differentially-expressed as a function of its degree of differential expression. Line types represent three different differential expression magnitudes: 2-fold (dotted line), 4-fold (dashed line) and 16-fold (solid line). A gene was defined as detected if it was assigned a Benjamini-Hochberg corrected P-value for the difference in group means of less than 0.01.

To illustrate the performance of a simple conventional analysis method, we applied a t test to the simulated data. The t test displayed a rapid increase in detection performance with increasing degree of differential expression, with the critical degree at which detection became likely varying with the sample size, magnitude of differential expression, and supplied test size (Figure 2). Notably, manipulation of the one free parameter in the t test (the test size) did not effectively change the degree of differential expression required for consistent detection (data not shown).

In contrast to the t test, Messina could be tuned to detect low frequency aberrant expression. However, it was important to verify that this improved flexibility did not come at the expense of specificity, and that Messina was still strongly selective against genes that did not satisfy its supplied criteria. Messina's specificity naturally increases as the minimum degree of differential expression required for detection is increased. In simulations of data with 20 samples per group, Messina's false discovery rate (FDR), defined as the fraction of detected genes with less than the minimum required simulation frequency of differential expression, was 2.26%. For the more demanding case of only five samples per group, Messina's FDR was 8.17% at the very liberal 50% sensitivity cutoff, and only 1.09% at the more stringent 90% cutoff. In all cases, even under demanding conditions of a very liberal minimum classifier sensitivity cutoff and small sample size, Messina effectively controlled the false discovery rate.

### Illustration of Messina vs limma

Simulation studies indicated that Messina reliably detected inconsistent differential expression in complex microarray datasets with few samples per group. In order to demonstrate its relevance to contemporary biological problems, we compared the algorithm to limma, an established conventional analysis platform, in the analysis of a representative experiment. Both Messina and limma were used to analyse a previously-published microarray data set [8] comparing human pancreatic cancer samples to normal pancreas.

**Figure 3:**
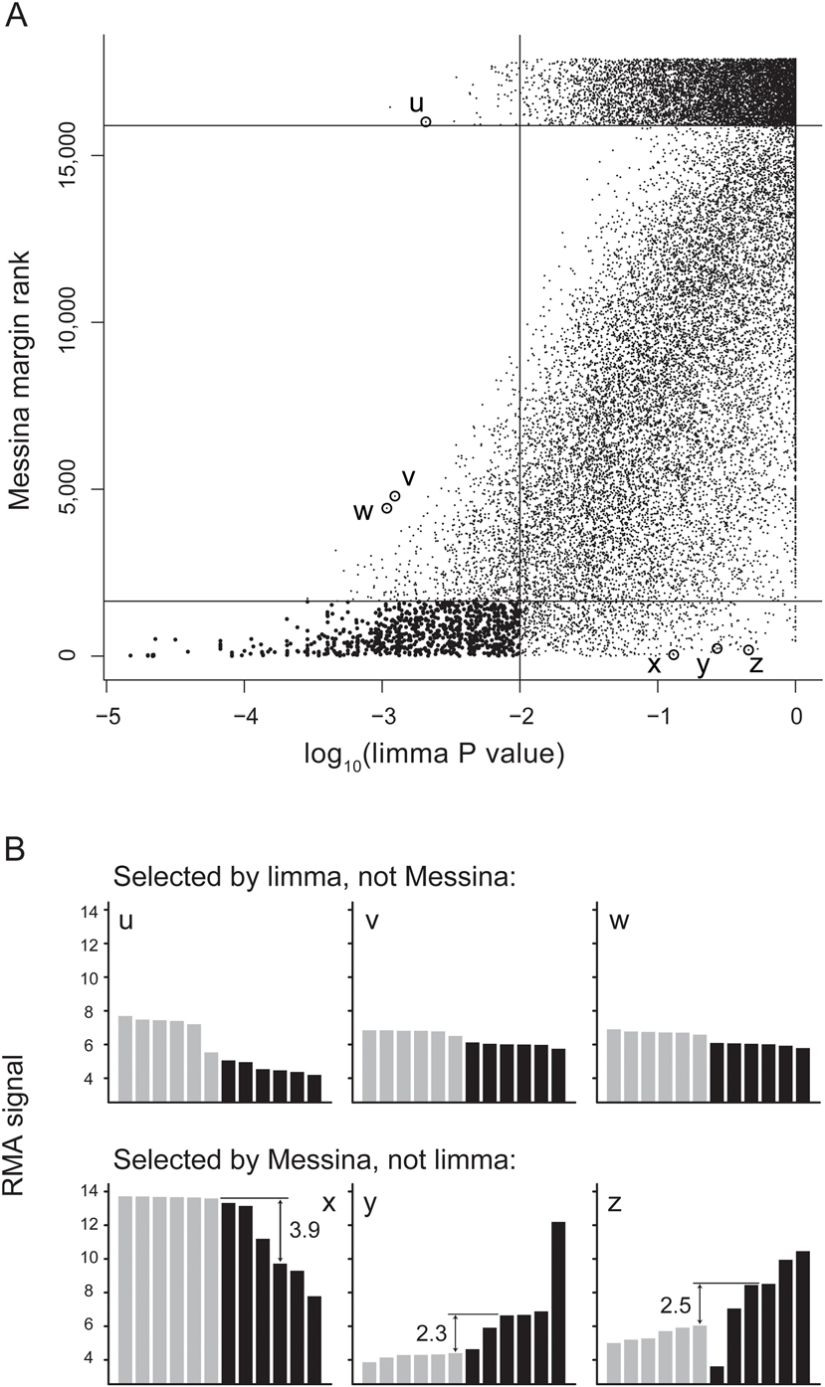
Comparison of Messina and limma results. (a) Log_10_-transformed limma P-values are displayed on the horizontal axis; a vertical line at −2 units denotes the P<0.01 cutoff value. Ranks of Messina-assigned margins occupy the vertical axis, with larger margins being assigned smaller ranks; a horizontal line at rank 1,646 denotes the margin >1 cutoff value. Limma P-values for probesets rejected outright by Messina (ie. no threshold could satisfy the input performance requirements) are displayed above the horizontal line at rank 15,898; the vertical position for these Messina-rejected probesets has been jittered for display purposes only. Probesets selected by both methods are plotted as filled black circles, all other probesets are plotted as black dots. Six discordant probesets that were selected for closer examination are highlighted by open circles. (b) Expression profiles of selected discordant probesets. Grey and black bars represent normal and cancer samples, respectively. For the probesets selected by Messina (panels x−z), the relevant feasible region and margin size are marked.

The results of the Messina and limma analyses were broadly concordant (Figure 3a). Of 44,928 probesets in total, 42,405 (94.4%) were considered not differentially expressed by either technique, while 837 (1.9%) were considered differentially expressed by both techniques. The techniques disagreed for 1,686 (3.8%) probesets, 809 of which were considered differentially expressed by Messina alone, and 877 of which were only considered differentially expressed by limma.

To demonstrate the differences between the analysis techniques we selected six illustrative cases of the 1,686 discordant probesets (Figure 3b). All three Messina-selected probesets displayed a high intra-group variability, with one to two cancer samples exhibiting an expression level close to that of normal tissue (Figure 3b, panels x–z). Nonetheless, all three probesets could form the basis of single-gene classifiers with wide classifier margins. Conversely, two of the probesets selected by limma, but considered unattractive by Messina, show quite low intra-group variability and a small mean difference (Figure 3b, panels v, w). A third probeset selected by limma was rejected outright by Messina (Figure 3b, panel u). Although this probeset could be used to construct a classifier with a reasonably large margin, the presence of a sample of normal tissue with expression close to the cancer group drastically reduces the specificity of a classifier based upon this probeset. As Messina was supplied a minimum specificity requirement of 90% when these data were analysed, and this probeset could not satisfy this condition, it was rejected by Messina.

*S100A2* calcium-binding protein mRNA was detected as differentially-expressed by Messina, but not by limma (Figure 3b, panel y), and thus it was of interest to determine if S100A2 was differentially-expressed in a larger cohort, using a different assay to validate Messina's findings. S100A2 protein levels in a pancreatic cancer cohort of 115 patients [8] were measured by IHC. Scoring of staining intensity and percentage of staining cells revealed moderate or high S100A2 expression in 29 samples (25.2%), of which 13 samples (11.3%) displayed very strong staining, in agreement with the Messina results. High expression of S100A2 in this cohort was significantly associated with a poor prognosis (data not shown).

## Discussion

Genes that display low frequencies of differential expression in disease have often been neglected by high throughput studies despite their central role in the definition of molecular subtypes. Traditional analysis methods typically ignore such genes, and new techniques capable of identifying genes with low frequency aberrant expression have produced promising results [4], [5]. However, these outlier detection techniques offer little flexibility in the types of gene profiles selected, selecting only genes displaying high magnitude aberrant expression at a very low frequency. Messina is a flexible method that is ideally suited for the analysis of small datasets, and bridges the gap between traditional analysis techniques and newer outlier detection approaches. Messina's flexibility allows the user to input prior biological knowledge about the expected patterns of aberrant expression to inform the analysis, an advantage in heterogeneous diseases such as cancer.

Messina naturally detects low-frequency differential expression, an area upon which conventional analysis techniques do not specifically focus. When compared to the commonly-used t test, Messina was superior at identifying genes with low frequency differential expression (Figures 1,2), especially with small sample sizes, and maintained a low FDR under all conditions. Many more sophisticated analysis methods are refinements of the t test and share, to some degree, the penalty it applies to genes with low frequency aberrant expression.

When used for its designed purpose, Messina performed favourably when compared to a popular and highly powerful analysis platform, limma. The results of Messina and limma were broadly concordant (Figure 3); however their disparate results highlight their different application. Limma very sensitively detects consistent differences in expression, even when these differences are subtle, but permits little flexibility in the types of differential expression profiles found. Messina detects both consistent and inconsistent differences in expression and grants the experimenter great flexibility, but favours large margins. These approaches are complementary: limma and other conventional microarray analyses are appropriate to detect genes that are differentially expressed in close to every single sample of a group, whereas Messina is optimised to detect differences between groups even if the differential expression is present at a low frequency. Although Messina cannot analyse the complex experimental designs handled by limma, for a simple case-control type analysis it serves as a useful adjunct method.

The key strength of Messina is that the method provides a mechanism by which the user can control the types of gene profiles selected, and the performance and robustness of the classifiers that are based upon the selected genes. Messina accepts from the user the minimum sensitivity and specificity values that all classifiers (equivalently, genes) identified must satisfy, and therefore allows the experimenter to flexibly reduce the stringency of the gene selection process. The benefit of allowing more lenient cutoffs is twofold: in a classification context it permits the discovery of more robust classifiers, and in a gene discovery context it enables the detection of genes with inconsistent aberrant expression. If high classifier robustness or the detection of genes with inconsistent expression are a main goal, the sensitivity cutoff may be relaxed, trading classifier performance for improved resistance to noise, and a low false positive rate for improved detection of inconsistent aberrant expression. Should the analysis require it, the specificity cutoff can also be reduced, with a similar attendant trade-off. In simulations Messina faithfully matched the supplied constraints across a wide range of restrictions and gene expression scenarios, permitting the researcher to flexibly experiment with performance requirements with confidence that these constraints will be respected by the identified genes and classifiers. However, as the constraints are relaxed beyond a point the genes thus found will be progressively less likely to be biologically relevant; this point will vary with the particulars of the experiment and the downstream application of the identified genes, and defines Messina's lowest *practical* sensitivity and specificity thresholds. To our knowledge, no other analysis technique allows this control over the types of differential expression detected, nor the performance of classifiers thus generated.

Messina's ability to detect genes with inconsistent aberrant expression was demonstrated using immunohistochemistry, by which S100A2 was found overexpressed in 25% of samples. The expression pattern of S100A2 (Figure 3a, point y) is typical of markers of molecular subtype; such patterns are penalised by many conventional techniques due to their high intra-group variance.

Whereas conventional analysis techniques promise great sensitivity in detecting genes with subtle but consistent changes in expression, Messina offers the opportunity to identify those with inconsistent differences. Accumulated knowledge about disease biology indicates that both types of genes are likely to be important, and by using a combined approach the researcher is offered a broader range of analyses to guide further work. Messina's flexibility is unique in the emerging field of low frequency differential expression analysis, and this feature makes Messina a natural companion to traditional methods of analysis, being able to detect many different types of expression profiles depending on its input parameters. The Messina software is free, and includes a user-friendly graphical interface for use by biologists.

## Methods

### Threshold classifiers

The classifiers trained by Messina decide the class of an unknown sample by comparing the expression of a single gene in that sample to a threshold value. If the expression of an unknown sample exceeds the threshold value, the sample is assigned one class, and the remaining class otherwise. Threshold classifiers are completely specified by the classifier's threshold value, and its direction, the latter being the class to which samples with expression exceeding the threshold are assigned.

### Core training algorithm

Given the classifier direction and a set of sample expression values and associated classes, classifier sensitivity and specificity (as evaluated on the training data) are monotonic functions of the classifier threshold value. The (possibly empty) set of threshold values for which the sensitivity and specificity both satisfy the experimenter-supplied constraints is termed the *feasible region* for a given gene and classifier direction. Following the reasoning underlying the Support Vector Machine [9], the final threshold is selected as the midpoint of the feasible region, resulting in a maximum margin of safety before either of the classifier constraints is violated. This margin is employed as a measure of classifier robustness to noise: classifiers with larger margins are supposed to be more resistant to measurement error or peculiarities of the training set. Messina's training algorithm is illustrated in Figure S1 and described in more exhaustive detail in Methods S2, where the close link between Messina and empirical cumulative distribution functions is also developed.

### Messina implementation

For each gene, Messina's core training algorithm determines the feasible region for both classifier directions. In the case of both directions yielding a feasible region, the one with the largest margin is selected. In the case of genes that do not yield a feasible region for either classifier direction, the training algorithm produces a zero-rule classifier that randomly produces class labels with frequencies equal to the training set class frequencies. During cross-validation (CV), this naturally penalises genes which cannot consistently be used to produce acceptable classifiers.

Messina's core training algorithm is wrapped in a CV loop that employs independently-sampled data set splits [10]. The mean CV performance is compared to the performance constraints, and if these CV performance estimates satisfy the constraints, the classifier is trained on the full data set, and the full classifier parameters and CV performance reported to the user.

The overall steps of the Messina procedure are presented in pseudo-code form in Figure S2.

### Messina software

Software that implements the Messina algorithm is freely-available at http://www.garvan.unsw.edu.au/public/mpinese/messina.

### Detection performance simulation study

In order to test Messina's detection performance, we produced a series of simulated microarray datasets with known differential expression using data from a previously-published study [8]. This data set included six normal human pancreas samples hybridised to Affymetrix HG-U133A microarrays. To characterise the data for subsequent synthesis, array pre-processing was performed using RMA [11], and for each probeset a maximum-likelihood lognormal distribution was fit to the six normal sample RMA expression measures.

The synthetic datasets were generated by drawing simulated case and control samples from lognormal distributions: control samples from a distribution with a lower mean, and case samples from either the control distribution or a case distribution with a higher mean. The proportion of case samples drawn from the case distribution was varied to simulate a range of differential expression types from highly consistent (high proportion) to outlier (low proportion). Full details of the generation procedure follow. For each synthetic probeset, *n* simulated control samples and *n* simulated case samples were generated by random sampling from two lognormal distributions. The mean of the lognormal distribution of the *n* control samples, and *m* of the case samples, was ln *t*; the mean of the lognormal distribution of the remaining *n*–*m* case samples was ln (*t* + δ), with δ representing a gene's magnitude of differential expression. In the experiments, *t* ∈{4, 6, 8, 10}, δ ∈ {1, 2, 4}, *m* ∈{0, …, n}, and *n* ∈{5, 10, 20}. *m* represents the number of case samples that exhibit a control-like distribution; as *m* approaches *n* the proportion of simulated case samples that are generated from the case distribution decreases. To simulate the heteroskedascity common in array data, standard deviations of the simulated probeset lognormal distributions were generated empirically by random sampling from the standard deviations of the lognormal fits to the array data. This sampling was performed independently for the samples with mean ln *t* and the samples with mean ln (*t* + δ); sampling was from within those fitted probesets that had a fitted mean expression value within 0.1 percentiles of the simulated probeset mean. A simulated gene's degree of differential expression was defined as *d* = 1−*m*/*n*. 100 simulated probesets were generated for each combination of *t*, δ, and *n*, yielding 45,600 simulated probesets in total.

The Messina analysis and a two-sample t test procedure were applied to the simulated data in order to evaluate the detection performance of the two methods. Messina was run upon the data three times, with sensitivity cutoffs of 50%, 70% and 90%; all runs employed a specificity cutoff of 90%, 60 CV iterations, and a training set size of 90% of the full data. A probeset was considered detected by Messina if its CV sensitivity and specificity were both at least as high as the supplied cutoff values. Welch's two-sample t test was also applied to the data, testing for each probeset the null hypothesis of no difference between the case and control means. A probeset was considered detected by the t test if its Benjamini-Hochberg corrected [12] P-value was less than 0.01. Detection probability was defined as the proportion of probesets that were detected by an analysis method for fixed values of δ and n, as a function of the degree of differential expression.

### Application to pancreatic cancer data

In order to contrast Messina with conventional techniques, we applied the algorithm to a small pancreatic cancer data set, and compared its performance to that of an established microarray analysis technique [13] implemented in the package limma [14]. A subset of a previously-published microarray study [8] was used; this subset featured data from six bulk pancreatic adenocarcinomas and six unmatched bulk normal pancreata, hybridised to Affymetrix U133 A and B arrays. Array pre-processing was performed using RMA [11].

The array data were analysed separately using Messina and limma. In the Messina analysis genes were selected if their CV performance passed the supplied performance thresholds of 50% sensitivity and 90% specificity and they had a classifier margin of at least one log_2_ unit; in the limma analysis genes were labelled as differentially expressed if they were assigned a Benjamini-Yekutieli (BY) corrected [15] P value for no difference between cancer and normal mean expression of less than 0.01. To facilitate comparison between Messina and limma results, the rank of Messina's reported probeset classifier margin (a measure of the robustness of the trained classifier) was compared to the log_10_-transformed BY-corrected [15] P value reported by limma.

### Immunohistochemistry

To validate Messina's results, we measured expression of S100A2 protein by immunohistochemistry upon a separate patient cohort from that used in the microarray data [8]. Tissue microarray sections were dewaxed in xylene and rehydrated, then unmasked in retrieval solution (S2367, DAKO) at 121°C in a pressure cooker for 5 minutes. Slides were blocked with 3% H_2_O_2_ in methanol and incubated with 1:50 mouse antibody to S100A2 (DAK-2100A2/1, NeoMarkers) for 60 minutes. Detection was performed using Envision+ anti mouse (DAKO) with a 3,3′-diaminobenzidine substrate and slides were counterstained with hematoxylin. All slides were double scored by independent observers including a histopathologist.

## Supporting Information

Methods S1 Informal development of the reasoning underlying the core of the Messina algorithm.
(0.03 MB DOC)

Methods S2 Formal development of the link between the Messina algorithm and inverse eCDF estimation.
(0.06 MB DOC)

Figure S1 Illustration of the Messina training algorithm. The main plot shows the classifier sensitivity or specificity as a function of the threshold, for the classifier direction in which expression less than the threshold value is associated with control samples. Sample expression (n = 6 per group) is depicted by boxes beneath the main plot; empty boxes represent control samples and filled boxes case samples. The algorithm's supplied performance limits in this example (sensitivity ≥ 0.6, specificity ≥ 0.9) are represented by horizontal dotted lines, and when combined with the sensitivity and specificity curves define threshold values that produce classifiers with acceptable sensitivity and specificity, respectively. The range of possible thresholds in which both sensitivity and specificity satisfy the supplied constraints is denoted the feasible region. Messina selects a threshold in the centre of this feasible region, and defines the classifier margin as the width of the feasible region.
(0.82 MB TIF)

Figure S2 The Messina algorithm pseudo-code.
(0.03 MB DOC)
