# Supplementary material for: Messina: A Novel Analysis Tool to Identify Biologically Relevant Molecules in Disease
Source: PLoS One. 2009 Apr 28;4(4):e5337. doi: 10.1371/journal.pone.0005337 (PMC2671167; doi:10.1371/journal.pone.0005337)
Supplement: Methods S1 — Informal development of the reasoning underlying the core of the Messina algorithm. (0.03 MB DOC) [file pone.0005337.s001.doc]

Messina recasts the problem of identifying genes with low frequency aberrant expression into a classifier building problem. This section develops in more detail the equivalence between these two approaches.

Consider a single gene which has been measured across a number of samples that fall into two groups, case and control. Suppose that each group has an associated *canonical expression* level (expression level around which most of the samples of that group fall, with some small error), but that some samples display a level of expression close to the canonical level of the *wrong group*. Each sample can be described as having a case-like or a control-like expression level, depending on which canonical level is closest to the sample’s measured level. This situation is illustrated below:

**Illustration of low-frequency aberrant expression. The hypothetical expression of five control samples (grey) and five case samples (black) is shown. Most samples display a level of expression consistent with their group (case or control) with some small additional error, but some exhibit a level of expression consistent with the wrong group. The presence of these exceptional samples reduces the consistency of the differential expression between the two groups.**

Expression

Case samples

Control samples

Control-like expression

Case-like expression

Aberrant samples are a common feature of disease biology, particular cancer. In cancer, normal samples exhibiting tumour-like expression may indicate genes involved in early carcinogenesis or pre-cancer processes, and tumour samples with normal-like expression may define molecular subtypes, or may be a natural consequence of the heterogeneity of cancers. In all cases, genes with such patterns of expression should not be discarded out of hand.

The situation described above can be characterised by three values: the proportion of case samples with case-like expression (herein termed *p*), the proportion of control samples with control-like expression (*q*), and the difference between the canonical expression levels. If the random error by which a sample deviates from its canonical level is sufficiently small, then a threshold classifier with threshold equal to the midpoint between the canonical expression levels can determine whether a sample shows a case-like or control-like expression level, with perfect accuracy. If such a classifier is used to classify each sample into case or control groups based upon whether it displays case-like or control-like expression, then the classifier’s measured sensitivity is equal to *p* and its specificity is equal to *q*.

In this way the performance metrics of a threshold classifier can act as surrogates for the key values characterising a gene with an aberrant pattern of differential expression. By using common machine learning techniques such as resampling, it is possible to determine with some confidence the performance metrics of a classifier constructed from a given gene, and therefore the values characterising the pattern of differential expression. Messina employs this equivalence between the model of expression developed above and the performance of threshold classifiers to identify those genes with a desired pattern of aberrant expression, ranging from no aberrant expression whatsoever, through to very high levels of aberrant expression.
