# Supplementary material for: Messina: A Novel Analysis Tool to Identify Biologically Relevant Molecules in Disease
Source: PLoS One. 2009 Apr 28;4(4):e5337. doi: 10.1371/journal.pone.0005337 (PMC2671167; doi:10.1371/journal.pone.0005337)
Supplement: Methods S2 — Formal development of the link between the Messina algorithm and inverse eCDF estimation. (0.06 MB DOC) [file pone.0005337.s002.doc]

Considering the expression of only one gene, let $X_{i}$ be the expression values for samples *i* = 1, 2, …, *n*. The samples fall into two groups (eg. case and control), denoted 0 and 1, with $C_{k}$ the set of indices of the samples in group *k*, for *k* = 0, 1.

For simplicity we initially develop Messina assuming that larger expression values are associated with membership of group 1. Given a sample $X_{i}$ and classifier threshold *t*, Messina’s classification rule assigns a predicted group $\hat{k}_{i}$ as $\hat{k}_{i}=I(X_{i}>t)$, where $I(A)$ is the indicator of event *A*. When evaluated on the training data $X$, the realised classifier sensitivity $R_{N}$ and specificity $R_{P}$ are given by and .

Classifiers found by Messina are required to satisfy the performance constraints$R_{N}\geq S_{N}$ and $R_{P}\geq S_{P}$ when evaluated on the training data, where $S_{N}$ and $S_{P}$ denote the minimum acceptable sensitivity and specificity, respectively, as input by the user; $S_{N}+S_{P}>1$. These criteria either cannot be satisfied for any value of *t*, or will be satisfied by a single interval of threshold values, $t\in\left[T_{L}, T_{U}\right]$. is used as the final selected threshold, being the value of the threshold most distant from violation of either of the performance constraints. In this way Messina builds maximum-margin classifiers, with margin equal to $M_{F}=T_{U}-T_{L}$.

The central task of the Messina training algorithm is to find $T_{L}$ and $T_{U}$, given *X*, *C* and *S*. This is efficiently performed by restating Messina in the framework of empirical cumulative distribution functions (eCDFs). The eCDF for group *k* is given by $F_{k}(x)=\frac{1}{\left|C_{k}\right|}\sum_{i\in C_{k}} I(X_{i}\leq x)$, and its corresponding inverse by $Q_{k}(p)=\inf\{x:F_{k}(x)\geq p\}$. In this framework, $T_{L}=Q_{0}(S_{P})$ and $T_{U}=Q_{1}(1-S_{N})$. If $T_{L}>T_{U}$ then no value for the threshold yields a classifier with the required performance (determined by *S*) when evaluated upon the training set, and the training fails.

For each gene Messina attempts to train two classifiers – one assuming that higher expression is associated with case samples (as was developed above), and the other assuming that higher expression is associated with control samples. The latter is simply implemented by multiplying all expression measurements by ­–1 and repeating the classifier training, inverting the final threshold at the end. The classifier with the widest margin of the two created is selected as the final candidate; if neither produces a suitable margin the training fails.
