# Supplementary figures and images for: Messina: A Novel Analysis Tool to Identify Biologically Relevant Molecules in Disease
Source: PLoS One. 2009 Apr 28;4(4):e5337. doi: 10.1371/journal.pone.0005337 (PMC2671167; doi:10.1371/journal.pone.0005337)

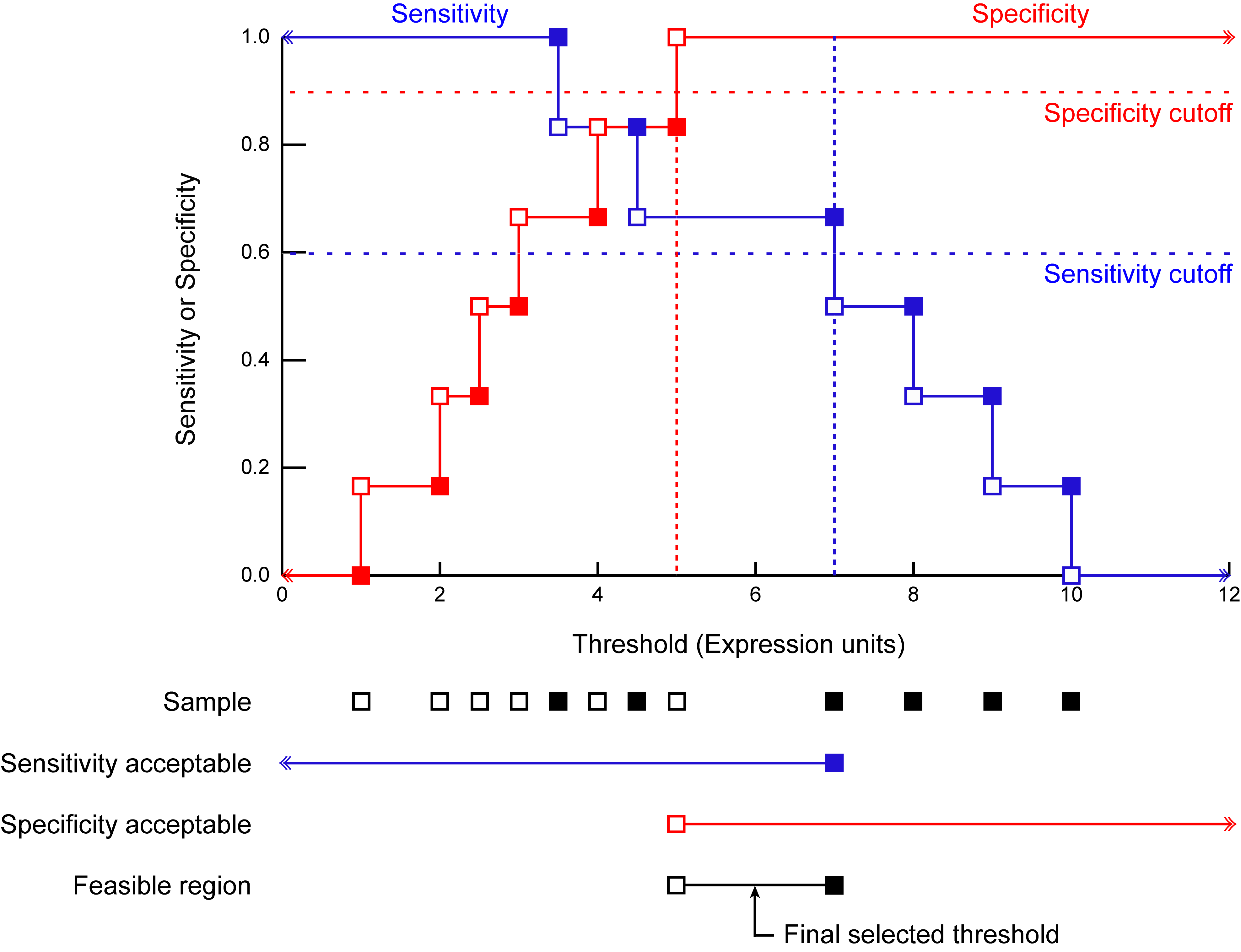

Supplement: Figure S1 — Illustration of the Messina training algorithm. The main plot shows the classifier sensitivity or specificity as a function of the threshold, for the classifier direction in which expression less than the threshold value is associated with control samples. Sample expression (n = 6 per group) is depicted by boxes beneath the main plot; empty boxes represent control samples and filled boxes case samples. The algorithm's supplied performance limits in this example (sensitivity ≥ 0.6, specificity ≥ 0.9) are represented by horizontal dotted lines, and when combined with the sensitivity and specificity curves define threshold values that produce classifiers with acceptable sensitivity and specificity, respectively. The range of possible thresholds in which both sensitivity and specificity satisfy the supplied constraints is denoted the feasible region. Messina selects a threshold in the centre of this feasible region, and defines the classifier margin as the width of the feasible region. (0.82 MB TIF) [file pone.0005337.s003.tif]
