# Supplementary material for: Messina: A Novel Analysis Tool to Identify Biologically Relevant Molecules in Disease
Source: PLoS One. 2009 Apr 28;4(4):e5337. doi: 10.1371/journal.pone.0005337 (PMC2671167; doi:10.1371/journal.pone.0005337)
Supplement: Figure S2 — The Messina algorithm pseudo-code. (0.03 MB DOC) [file pone.0005337.s004.doc]

§

1. For each feature (e.g. gene) in the data set:
   1. For each cross validation iteration:
      1. Randomly split data into training and test sets.
      2. For each of the two classifier directions:
         1. Find the feasible region, if any.
         2. Calculate and save the classifier margin.
      3. Were any feasible regions found?

Yes: Base the threshold and direction on the feasible region with the largest margin.

No: Mark this training instance as a failure, and adopt a zero-rule classifier for later performance testing.

- - 1. Evaluate the performance of the trained classifier on the test set, and save performance results for this cross-validation fold.
  1. Take the mean of all cross-validation fold test set results to yield cross-validated final performance estimates for this feature.
  2. Does the feature satisfy the performance constraints, as evaluated on the cross-validation results?

Yes: Train the classifier as above on the full data set for this feature, and output the full classifier and cross-validation results for this feature.

No: Discard this feature.

2) Report results.
